# Subjective Motives for Requesting In-Patient Treatment in Female with Anorexia Nervosa: A Qualitative Study

**DOI:** 10.1371/journal.pone.0077757

**Published:** 2013-10-25

**Authors:** Pauline Gorse, Clementine Nordon, Frederic Rouillon, Alexandra Pham-Scottez, Anne Revah-Levy

**Affiliations:** 1 Department of Adult Psychiatry, CMME, Sainte-Anne University Hospital, Paris, France; 2 INSERM 669 research unit, Paris-Sud University and Paris-Descartes University, Paris, France; 3 Argenteuil Hospital Centre, Argenteuil, France; University of Leicester, United Kingdom

## Abstract

**Background:**

Anorexia nervosa is a severe psychiatric disorder mainly affecting women. Its treatment is long and accepted with much difficulty, in particular in-patient treatment.

**Aims:**

To describe the subjective motives of women with anorexia nervosa for requesting in-patient admission, from a qualitative analysis of application letters.

**Methods:**

Participants were adult women (18 years and older) with anorexia nervosa who were admitted as in-patients in a referral hospital unit in France from January 2008 to December 2010. The application letters, prerequisites to admission, were studied by the interpretative phenomenological method of content analysis.

**Results:**

63 letters have been analysed, allowing the identification of six themes related to requests for in-patient care: loss of control of behaviour, and of thoughts, mental exhaustion, isolation, inner struggle and fear of recovery.

**Conclusions:**

Requests for in-patient admission were motivated by very personal, subjective experiences, unrelated to medical reasons for admission. These results may help improve pre-admission motivational work with individuals, by basing it on their subjective experience.

## Introduction

Anorexia nervosa is a serious disease with a lifetime prevalence currently estimated at 0.3% in teenagers [1] 2.2% among young adult women [2] and possibly rising [2]. It leads to numerous physical and psychiatric complications, especially suicide [3]. Its mortality rate has been estimated to exceed 5% [4]. Fewer than half of all patients recover, and the rate of chronic anorexia is estimated at around 20% [5]. Treatment of anorexia nervosa is thus a major issue in medicine. Current treatment guidelines in anorexia nervosa recommend multidisciplinary management in specialised centres, combining nutritional rehabilitation and psychosocial interventions [6]. First-line treatment is based on outpatient care [6,7] but inpatient treatment should be considered in situations of medical or psychological threat such as suicidality [7], or if the disorder has not improved with ‘appropriate outpatient treatment’ [6]. As a whole, the ‘patient’s overall physical condition, psychology, behaviours, and social circumstances’ should be considered when choosing a treatment setting [8]. Individuals with anorexia nervosa frequently refuse treatment [9]. Some studies have examined the factors associated with treatment acceptance, especially in specialised in-patient treatment centres. Halmi et al [10] suggest that among people with anorexia nervosa, a high level of obsessive preoccupations, assessed by the Yale-Brown-Cornell Eating Disorder scale, is a predictive factor for the acceptance of care involving cognitive-behavioural therapy, with or without treatment by fluoxetine. Compared with patients with purely restrictive anorexia nervosa, those with bulimic anorexia or purging behaviour may accept in-hospital treatment more easily, according to Tasca et al [11]. The same appears true for patients with depressive symptoms or strong body dissatisfaction, independently of type of anorexia nervosa. These studies are important, but the factors they measure are clinical and assessed by the medical team. They do not consider the subjective experience of patients who agree to or request in-patient treatment. Recently, Paulson-Karlsson et al [12] used qualitative methods to determine what patients with anorexia nervosa expected of specialised treatment. The expectations were high and the patients hopeful, but the study does not provide information on what may have triggered the request for help. 

The aim of our study was to identify the reasons for requests for in-patient care by women with anorexia nervosa, from their own point of view. To do so, qualitative methods have been used aiming at describing, understanding and detailing observed phenomena. Qualitative methods are a tool of choice for focusing on the patients’ perspectives and are thus increasingly used in research on this disease [13].

## Materials and Methods

### Participants

The Clinique des Maladies Mentales et de l’Encéphale (CMME) at Sainte-Anne Hospital, Paris, France, is an adult psychiatric department providing specialised care for Eating Disorders (ED) and comprising an outpatient and an in-patient ED unit. Patients may be referred to this department by their psychiatrist, their general practitioner or even themselves. They may come from metropolitan or overseas France.

Our study sample was a consecutive series of: (a) female patients, (b) aged at least 18, (c) with anorexia nervosa as defined by the Diagnostic and Statistical Manual of Mental Disorders, fourth edition [14] and (d) admitted to the ED in-patient unit between January 2008 and December 2010.

Our sample consisted of 63 women with anorexia nervosa, 40 with anorexia nervosa restrictive type and 23 with anorexia nervosa binge-eating/purging type. Mean body mass index at admission was 14.7 kg/m^2^ (SD = 7.0). Their mean age was 27.6 years (SD = 9.0). Fifty women (82%) were unmarried, and only 5 had children. In all, 27 (42.9%) were students, 16 (25.4%) had jobs, and the others were looking for work or on sick leave.

### Ethics Statement

For the present study, all the data used have been collected in the normal course of care and as a part of the regular admission procedures. Patients were informed that data were to be used in future studies and verbal consent was obtained, which was enough to fulfil requirements from the French Law on Bioethics. Also, data computerization was approved by the French commission on data computerization and personal freedoms (Comité National Informatique et Libertés, CNIL) and The French Ethics Committee for medical research (Comité Consultatif sur le Traitement de l’Information en matière de Recherche dans le domaine de la Santé, CCTIRS).

### Measures

#### Application letters

All patients refereed to the CMME for inpatient care in the ED department have a pre-admission medical interview and need to write an ‘application letter’, prerequisite to this pre-admission medical interview. The purpose of this letter is (a) for the patient, to introduce him/herself and explain his/her reasons for wanting in-patient care and (b) for the staff, to detect any emergency. The instruction for this letter is provided by a specialized nurse team and is a broad instruction: ‘Please explain why you want to be admitted to our ED unit and state your weight and height’. At the pre-admission interview, the letters are included in the patients’ medical records.

#### Socio-demographic and clinical data

On admission to the ED hospitalisation unit, basic socio-demographic and clinical data were collected, as described elsewhere [15].

### Data analysis

Application letters were extracted from medical files and provided, without any names attached, to the research team for qualitative analysis. Sample size was defined by criteria of data saturation.

NVivo 9 qualitative data analysis software [16] was used to assist our data management and analysis. All letters were analysed by interpretative phenomenological analysis (IPA), with word-processing and spread sheet software. Using this approach, no pre-existing theme is imposed on the data but themes begin to emerge as the analysis takes place. The data analysis procedure was thus inductive. The literature was reviewed after this step. First, two authors (PG and ARL) specialized in qualitative research, repeatedly read the letters (in their original language, i.e. French). Concept codes were developed and initial themes were identified and annotated in margins. This was close to being a free textual analysis. Each reading of the letters had the potential to bring up new insights. Second, recurrent themes, defined as themes reflecting a shared understanding of the phenomena in question among participants [17], were identified across letters. This second step involved a more analytical ordering, as researchers tried to provide a meaning to the connections between themes. Some of the themes tended to cluster. The aim of this dynamic and cyclic process was to recognize ways in which participants’ narratives were similar but also different. Thus researchers integrated new emerging themes, to take convergences and divergences in the data into account [18]. 

To enhance validity, the analysis was carried out by two authors (PG and CN). Moreover, the letters were read by one other author (ARL) to improve consistency and coherence of the IPA. The research team met repeatedly, to ensure that the themes identified were an accurate reflection of the data and that the analysis was not confined to a single perspective. The meetings helped to highlight necessary clarifications and modifications among the themes identified [18]. 

Extracts of letters have been selected and are provided below, in order to exemplify each underlying theme. The letters have been freely translated into English for the sole purpose of this article. This translation tried to preserve the essential meaning and content of the letters and, as far as possible, their general tone.

## Results

Saturation of themes was obtained after the reading of 63 letters, corresponding to 63 patients. All the letters were hand-written, on standard size sheets. Their mean length was 2.3 pages (SD = 1.0) with a range from 1 to 5.

Although the instructions about the letter content were quite vague, most women described the history of their disease in a chronological order. This description was factual and matched the clinical history of anorexia nervosa. Accordingly, this was not analysed it in terms of content.

Six principal themes were identified as being associated with the request for in-patient care and were categorised in three domains of experience: loss of control, intensity of distress, and ambivalence.

### Loss of Control

The participants described the transition from the impression of control their disease initially conferred to the loss of control that followed. The participants explain that at the beginning of the disease, this feeling of control made them feel valuable and that progressively, they came to realise that it was illusory. In the letters, the expressions ‘vicious circles’ and ‘infernal spirals’ recurred frequently.

#### Loss of control of their behaviour

Food was described as omnipresent in their behaviour (dietary restrictions, bulimic episodes). Women described a feeling of being ‘possessed’, as if someone else was acting in their place and if they no longer felt like the same person.

‘I don't want to live anymore with this suffering, with this control that's over me, food, which runs everything, which is destroying me, possessing me.’ (Letter 9)

‘It's as if my body has taken power, I'm no longer in control of anything...’ (Letter 36)

Bulimic episodes, when present, seemed to be an extremely distressing symptom. The women described themselves as inside their ‘bubble’ with their own reality constructed around these episodes of bingeing and vomiting.

‘My life is made up of bulimic crises with anorexia in between; it has become a permanent ritual.’ (Letter 54)

‘I spend my time eating too much, and badly and too fast, until I'm nauseated and I vomit it up. Then when I have nothing more to vomit, naturally I drink tons of water or some other liquid, to have a full stomach and have something to throw up, and that makes me feel less bad. I can't control myself, I have to fill myself up to empty myself afterwards.’ (Letter 55)

#### Loss of control over thoughts

In their letters, the women described a world made up of food obsessions so invasive that they left no room for anything else. Sometimes, the letters talked only about food, with a list of several pages of what the writer ate, how she calculated what she would allow herself to eat as a function of her meals and daily exercise. They described their minds as totally invaded, devoured, by the obsession with food.

‘I am obsessed by food, I have the impression that I'm wearing glasses made of food, because my only concern is to know what I should do, if I should eat it.’ (Letter 59)

‘Obsession with food, this subject is literally devouring my mind.’ (Letter 11)

‘I can't stand it anymore! This time I'm starting to realise that I'm lost… I cannot get these thoughts focused on food out of my head, they invade me and won't leave me alone … The only moment I don't think of it is when I actually start to do it [to binge]! And even when I'm throwing up, I sometimes start thinking about the "next time", organising it in my mind!’ (Letter 46)

### Intensity of distress

The women described the intensity of their suffering and mental exhaustion. Another aspect of their distress was the extreme loneliness.

#### Exhaustion

The participants reported that their suffering was unbearable and combined with real exhaustion; in some letters, this was expressed as morbid thoughts.

‘Disgust with myself, endless suffering… it is awful when you cannot even recognise yourself …  can't stand yourself, who you are, the struggles, the continual losing battles to binges.’ (Letter 47)

‘I don't recognise myself anymore. If I try to take stock of these last two years, I realise how much I've changed: I have no taste for anything, especially for living. I'm very weak and become exhausted easily. I get irritated constantly; I'm pessimistic and get depressed a lot…’ (Letter 44)

‘It's eating me up, destroying me, a kind of slow suicide, little by little…’ (Letter 26)

#### Isolation

The women explained that their disease cut them off from the outside world, made up of their family, their friends and their work. On the one hand, the constraints imposed by the disease made relationships with others extremely complicated. On the other hand, the women explained that they retreated into themselves or could be aggressive or even tyrannical with those around them. They also described their family's worries, but their perception of this concern was not one of their motives for seeking care. Those who worked explained that the physical consequences of their malnutrition forced them to stop working. They thus progressively found themselves very lonely.

‘Today my entire life is a disaster: I have no social life, no friends, just my family who supports me. I can’t stand it anymore, because I see that over time my behaviour has deteriorated. I’m tyrannical with my poor parents, I make them suffer, I harass them with my eating problems. […] I’m shut in, a prisoner of my obsession. I make myself suffer horribly and I make my parents suffer horribly. I am destroying them by harassing them at each meal and in always asking them if they haven't given me too much. I’m mean to them, aggressive. I get so angry at myself for being like this ... It’s me who’s fiendish, but I cannot make myself change.’ (Letter 16)

‘I’m stuck in this disease that makes my whole life (work, social, relations…) totally non-existent.’ (Letter 62)

‘It’s almost two years since this wretched thing has taken over my life, destroying me, cutting me off from the world, from my family and from life. It has made me disgusted with this precious thing that is life.’ (Letter 57)

### Ambivalence

Awareness of ambivalence seemed to encompass two aspects: inner struggle and fear of recovery. These two themes, although distinct, may also be interconnected. That is, inner struggle may reinforce the fear of recovery and vice versa.

#### Inner struggle

In describing ambivalence, women used the ideas of inner duality, of tugging. They described themselves as constantly pulled between two opposing forces: on the one hand, the desire for recovery and to gain weight and, on the other hand, the desire to restrict themselves, to burn up the calories ingested. In this unending battle, they appear lost and outmatched.

‘There’s two parts of me. One wants to get out of here, cries for help and asks for help, and the other one doesn’t see any problem. So much so that the delusion part is winning and that food has become my enemy… This part of me, which forbids me to have anything, is stronger than everything…’ (Letter 60)

‘It’s true that I am very unstable. Suddenly, I can’t stand it anymore, all that. I try to motivate myself, and then the next day, the fear of getting fat, of eating too much, comes back in force and I restrict myself or exercise myself to death… I want all that to stop, for good, I want to be able to be happy, only I’m afraid for myself because I’m so ambivalent. I have the constant feeling that two beings are constantly struggling inside me: Marie and the anorexic.’ (Letter 16)

#### Fear of recovery

Another aspect of the ambivalence apparently lies in the fear of recovery, which represents the unknown for the patients and is described as agonising. Recovery would mean abandoning the disease and a part of oneself. The women explained that the disease causes great distress, but that initially it brought comfort: the disease is described as a solution to latent bad feelings, and women apparently found a new identity in it, that of an anorexic.

‘But at the same time this recovery terrifies me! It represents, I think, so many things, a huge evolution, an unknown, an enormous change, the end of an era, perhaps even mourning to be done. […] I’m afraid of the world, afraid of the future, I’m dying of fear! And at the same time, I want to quit this cocoon I’ve made for myself and that is keeping me from living my future…’ (Letter 25)

‘I’m afraid, I admit, of the unknown that is my future…’ (Letter 57)

## Discussion

### Main findings

In this qualitative study, 63 patient application letters have been used to identify the principal subjective themes associated with a request for in-patient care in a specialised treatment centre for anorexia nervosa.

Six principal themes have been identified; they can be regrouped into three domains of experience: perception of the loss of control of the symptoms, intensity of distress and awareness of ambivalence. These three domains of experience that together drive the women towards asking for help and more precisely for in-patient treatment reveal a process of awareness at three levels. First, the patients seem to be developing an awareness of their loss of control over their symptoms. This result is interesting because it echoes the need for control that characterises individuals with anorexia nervosa. The feeling of control that they perceived at the beginning of and throughout the disorder has progressively been replaced by a feeling of helplessness and impotence in the face of the disease. At this stage, the women appear to be able to ask for help, especially to regain this capacity for control. This result is in line with what Reid et al [19] have evidenced in a qualitative study of anorexia nervosa patients seeking for outpatient treatment: ‘the struggle to retain control resulted in some feeling desperate and suicidal, leading them to seek treatment’. The second level of awareness is this of the suffering and its intensity. Here, again, awareness of the distress linked to the disease echoes the initial phase of well-being engendered by weight loss. More generally, awareness of the suffering is the counterpoint to the initial positive self-reinforcement process, as Serpell et al showed [20], describing the major disease-linked benefits anorectic women initially feel, such as ‘feeling protected, gaining a sense of confidence, and feeling different’. Similarly, Nordbo et al [21] hypothesize that anorexia nervosa may procure a ‘feeling of stability, security and mental strength’ for women with this disease, thus enabling them to ‘avoid negative emotions’. Finally, the third dimension corresponds to the awareness of their ambivalence and specifically of their inner struggle. It appears that the patients realise they cannot cure themselves all alone, that they actually need specialised help and that this ambivalence makes outpatient care difficult. This awareness of the need for help again echoes the propensity of patients with anorexia to associate their desire for autonomy with doing ‘it all by themselves’. Indeed, anorexia nervosa often begins at a moment in their lives when young women begin to want to become autonomous. At the beginning of the disease, anorexia nervosa allows them to affirm themselves, to differentiate themselves from others and to create a distance from their family that gives them a feeling of independence [22]. Anorexia nervosa also has a role in providing an identity – the “anorexic identity” – as reported by Jenkins et al [23]. Patients who have recovered explain that the disease defined them as a person and that the recovery process implies the management and the change of this ‘anorexic identity’.

The three levels of this awareness thus conflict point by point with the initial experience of the disease and especially the feeling of control, the absence of distress or complaints, and the demand for autonomy and independence. Our study suggests that patients request in-patient treatment when the initial positive reinforcement and maintenance processes are no longer effective.

Another interesting result is that the reasons mentioned by the women for the request for in-patient treatment were eminently subjective and personal. Their request for admission to hospital was part of a personal process that seems to follow their personal experience of their increasing awareness. This result is the opposite of what has been suggested by earlier studies, which indicated that women agree to be admitted because they give in to pressure from their families or caregivers [24].

In addition, it could be noted a discrepancy between the medical priorities and the patients’ perspective. For the women, the decision to be treated as an in-patient appears to be focused on an internal process by which they become aware of their loss of control, of the intensity of their distress and of their ambivalence. On the other hand, the medical criteria for hospitalisation focus more on the physical emergency [8]. This discrepancy in physician and patient priorities has already been described by clinicians [25,26], but never previously demonstrated by the discourse of the women themselves. According to Guarda et al [25], individual motivation for treatment is linked to their desire to obtain a ‘moment of respite from the physical and psychological consequences of their disorders but without gaining significant weight’. The doctor-patient encounter is successful when they share the same priorities.

Finally, the absence of a theme related to the body itself is surprising and noteworthy. The body is approached only with medical terms, very factually, and solely to describe treatment. No emotion, no sensation is attached to the body. This distancing of the experience of the body and the emotions has been described in the literature. In particular, some authors suggest that disorders of self-perception may reflect a process of displacement to the body of unexpressed emotions or threatening feeling [27,28]. Moreover, this perceptual distortion is not frozen in time, but fluctuates as a function of the women’s emotional status and may be enhanced during difficult emotional situations [29]. If the perceptive distortions of the body enable negative emotions to escape, one can suppose that this symptom maintains the disorder and is therefore not a reason for a request for care.

## Strengths and Limitations

To our knowledge, this is the first time that the motives of women with anorexia nervosa for requesting in-patient admission have been studied, based on their own words. Moreover, this study includes a large number of patients and uses a rigorous qualitative methodology.

However, some limitations of this study must be pointed out. A first limitation is that only women were included, for there were too few men (four). Our results thus cannot be generalized to men or to minors, whose hospitalisation necessarily involves their parents. Another limitation to our results is the possibility of a ‘social desirability’ bias [30]. That is, the application letters were written for the purpose of obtaining access to specialised in-patient treatment for eating disorders. These letters thus had real stakes. One might think that some patients modified their discourse to convince the doctor to admit them. Nonetheless, the women’s discourse seems sincere, describing their universe openly to the point of sometimes giving the reader the uncomfortable feeling of reading a personal diary. Moreover, they mentioned their ambivalence and their difficulty in making this request for treatment; neither of these factors supports the idea that social desirability affected the discourse.

### Clinical implications

Our results may have several clinical implications.

First, our results emphasize the importance of motivational work with patients. Although motivational interviews were initially developed in the fields of substance abuse [31], they have a key role in the management of anorexia nervosa [32]. Their objective is to diminish ambivalence and resistance to change and to increase commitment to treatment [33]. During motivational interviews, clinicians use a ‘decisional balance’ tool, where the perception of the disadvantages in the current situation can engender a desire to change. Regarding anorexia nervosa, several studies have suggested that motivation for change is predictive of a better short-term prognosis [34,35]. Similarly, Espindola et al [36] report that ‘motivation and stimuli to remission’ are a major theme that emerges when patients in remission from anorexia nervosa are interviewed about it. The results of our study might allow clinicians to guide their interviews toward themes that are important for the patients, namely, loss of control, distress and ambivalence. Targeting these themes during interviews may help patients come to terms with their situation, at the moment when in-patient care becomes necessary. Moreover, the arguments advanced by physicians for hospitalisation could focus more on the patients’ subjective experience than on medical and somatic arguments.

A second valuable and practical aspect of knowing the patients’ own reasons for seeking in-patient care is to reduce the number of premature departures (dropouts) from specialised ED units. Premature departures account for 20 to 58% of admissions to these units [37] and have an increased risk of relapse at one year and of long-term chronicity [38]. One can suppose that having these individuals face their own motives for asking for admission signifies to them that they play an important role in their hospitalisation, which could have a positive effect on the continuation of care. This corresponds to the concept of ‘human agency’ [39], that is, people’s capacity to recognise themselves as the author of their acts, to attribute their ideas, actions, and feelings to themselves. Two previous studies have shown a low level of agency in patients with anorexia nervosa [40,41]. In-patient treatment could thus focus on helping patients perceive themselves as the agent of their treatment plan, with their own reasons for seeking this care.

In conclusion, requests for in-patient admission by patients with anorexia nervosa were found to be motivated by very personal, subjective experiences, unrelated to medical reasons for admission. These results may help improve pre-admission motivational work with individuals, by basing it on their subjective experience.
